# Modified specific gravity method for estimation of starch content and dry matter in cassava

**DOI:** 10.1016/j.heliyon.2021.e07450

**Published:** 2021-07-02

**Authors:** Kanvisit Maraphum, Khwantri Saengprachatanarug, Seree Wongpichet, Arthit Phuphuphud, Panmanas Sirisomboon, Jetsada Posom

**Affiliations:** a Department of Agricultural Engineering, Faculty of Engineering, Khon Kaen University, Khon Kaen, 40002, Thailand; b Applied Engineering for Important Crops of the North East Research Group, Faculty of Engineering, Khon Kaen University, Khon Kaen, 40002, Thailand; c Department of Agricultural Engineering, King Mongkut's Institute of Technology Ladkrabang, Bangkok, 10520, Thailand

**Keywords:** Cassava tuber, Starch content, Dry matter content, Breeding programme, Polarimetric method, Specific gravity

## Abstract

An empirical model for the estimation of starch content (SC) and dry matter (DM) in cassava tubers was developed as an alternative method to polarimetry and dry oven. These improved estimation equations were developed based on the specific gravity (SG) method. To improve accuracy, the one hundred-seventy-four sample were obtained from four commercial varieties of cassava in Thailand including KU50, CMR38-125-77, RY9 and RY11, respectively. The age of sample collected from four to twelve months after planting was used in this experiment. The empirical model was created from their relationships between SG obtained from small sample size (~100 g) and its SC and DM. The SG for cassava was strongly correlated with the SC and DM, with values for the coefficient of determination (R^2^) of 0.81 and 0.83, respectively. The SC showed a high correlation with the DM, with R^2^ of 0.96. To confirm that the empirical model was effective when applied to other samples, unknown samples collected from another area were tested, and the results showed a standard error of prediction (SEP) of 1.02%FW and 3.49%, mean different (MD) of -0.66%FW, -0.89% for the SC and DM, respectively. Hence, our empirical equation based on a modified SG method could be used to estimate the SC and DM in cassava tubers. It can help breeders to reduce costs and time requirements. Moreover, breeders could be used the methods to evaluate the SC and DM from the tuber formation to harvesting stage and monitoring the changes in SC and DM during breeding.

## 1 Introduction

Cassava (*Manihot esculenta Crantz*) is an important crop in economic terms, as it is the main raw material used for starch production. Cassava is considered a key crop in Thailand, which is the second-largest producer of cassava in the world and has the highest export value from cassava products (Johnson and Raymond, 1965). Cassava is particularly important as a source of starch in tropical and subtropical regions of the world. Approximately 85–90% of the total dry matter of cassava is starch content (SC) (Booth et al., 1976; OAE, 2018; Pomares-Viciana et al., 2018; Hatfield et al., 2014). SC is an important parameter indicating the quality of fresh cassava root. Climate change is now causing a reduction in the quality and yield of agricultural products (Mendelsohn, 2014; Arora, 2019), and the increase in the world's population means that the need for food, energy, and agro-production has increased (Arora, 2019). Cassava has therefore become an important raw material and is used to produce food, biofuel (ethanol), chemical products and animal feed (KURDI, 2015; Koopmans, 2005).

At present, an increase in the yield of cassava root or SC cannot be achieved by expanding the production plant area, since space is limited; however, an increased yield of cassava root and SC can be obtained by improvements in cultural practices, which is also a quite difficult. Even for the same variety of the plant, measures such as the starch content (SC), dry matter (DM), harvest index (HI) and yield production of cassava tubers can differ if they are planted in different conditions, such as in different areas, with different amounts of rainfall and so on. This means that suitable varieties and corresponding planting areas that can give high-quality yields should be identified, then it is essential to improve a specific variety always suited to the culture (Maraphum et al., 2020). Breeding programs are also important, since current varieties may give low productivity in the future due to changes in the weather, soil degradation and so on (Dahr, 2011). Cassava breeding programs may therefore be needed for the foreseeable future.

Currently, cassava breeding programs take a long time and require long experimental periods to obtain a satisfied results, since breeding requires pollination in order to produce hybrid varieties. Then, long period of time before cloned hybrid seeds can be obtained, each of which is assigned as a different variety. Each variety of seed is then planted and the best is chosen from among the hybrid populations (Huisenga, 2018; Mumby, 2018). The particular variety provides satisfactory values of SC, DM, HI and yield, it is selected. These indexes are used to compare the varieties developed in the trials, and especially the SC and DM for cassava root.

The idea of improving short-lived breeds is emerging, in order to support resilience to climate change. If a variety can provide a high SC accumulation rate and a consistent SC level over a significant period of time, it becomes a recommended variety. The variables of SC and DM are compared in terms of their relative values within the group of the population before being tested in the next process in the breeding program (Buddhakulsomsiri et al., 2018; Janket et al., 2018), i.e. in the phase of uniform yield trials (UYT). This is the final step in evaluating the best varieties. This process was carried out over two years. About 20 different selected strains were planted and examined, and the most promising varieties were released and shared with farmers (Ceballos et al., 2016).

During the breeding process, many cassava samples are collected from plants of different ages, planting areas, varieties etc., to measure the SC and DM. A method that can provide rapid, precise, and accurate results for the SC and DM is therefore required, to reduce the time involved. Several methods have been used to determine the SC for agricultural products, and particularly for tuber crops such as potatoes and cassava, and these include the 920.44 technique of the Association of Official Analytical Chemists (AOAC, 1995), the Krochmal and Kilbride (1996) method and the “ISO 10,520 Native starch - Determination of starch content - Ewers polarimetric method” of the European Economic Community (ISO, 1997; Commission Directive, 1999). Besides, the method is used to determine DM, it places the samples in the hot air over approximate 2–4 days or until weight constant.

The polarimetric method is used to determine the SC for cassava tubers, and requires a relatively small sample of approximately 100 g on a dry basis. This process required a time at least 4 days to achieve. However, this advantage is offset by the high cost of operation; for example, the sample must be placed in hot air oven at 60 °C for around four days, and is then mashed and sent to a laboratory, which takes a long time. An important aspect of this process is the use of chemical substances, which means that this method needs highly skilled labour.

Therefore, the alternative method used for the estimation of SC and DM in cassava breeding was the specific gravity (SG) method, presented by Wholey and Booth (1979), they studied the relationships between SC and SG; and DM and SG. They found that the SC and DM could be estimated where the SG value of any sample was known. The SG can be determined by weighing samples in air and in water. However, there was a requirement that the weight of cassava root samples could be at least 5 kg. Currently, breeder therefore measured SC of cassava root for one time at the harvesting stage (12 months after planting, 12 MAP) due to the need to obtain a sample that was large enough to estimate the SC across SG method. However, in the real situation the cassava does not harvest at only 12 MAP. It is starting to harvesting from 6-12 MAP. Hence, if users use the equation which developed for this method, it might be provided high error results.

Since SG method can be applied to smaller samples, this makes it possible to estimate the SC and DM. Therefore, the empirical equations for evaluating the internal quality of cassava from the initial stage (tuber formation phase) until the harvesting stage. The objective of this study was to develop an empirical equation that correlate the SC and DM to SG to predict the values of SC and DM for cassava tubers, from SG obtained from a small sample size (~100 g), and to compare the results with those of the polarimetric method and dry oven treatment, which samples were weekly collected from 4 to 12 months. Our predictive equation can be used as an alternative method to polarimetry and the dry oven technique. If the results are consistent, this will benefit cassava breeders in terms of reducing the costs and time required to develop new varieties of cassava. Moreover, breeders can measure the SC and DM during growth (between the age of 4 months after planting (MAP) until harvested time) and monitor the change of SC and DM during breeding. This can help breeding increase their possibility of success in terms of discovering a good varietying, and more importantly will reduce the cost and the time consumed in the breeding process.

## 2 Materials and methods

### 2.1 Sample preparation

**Figure 1:**
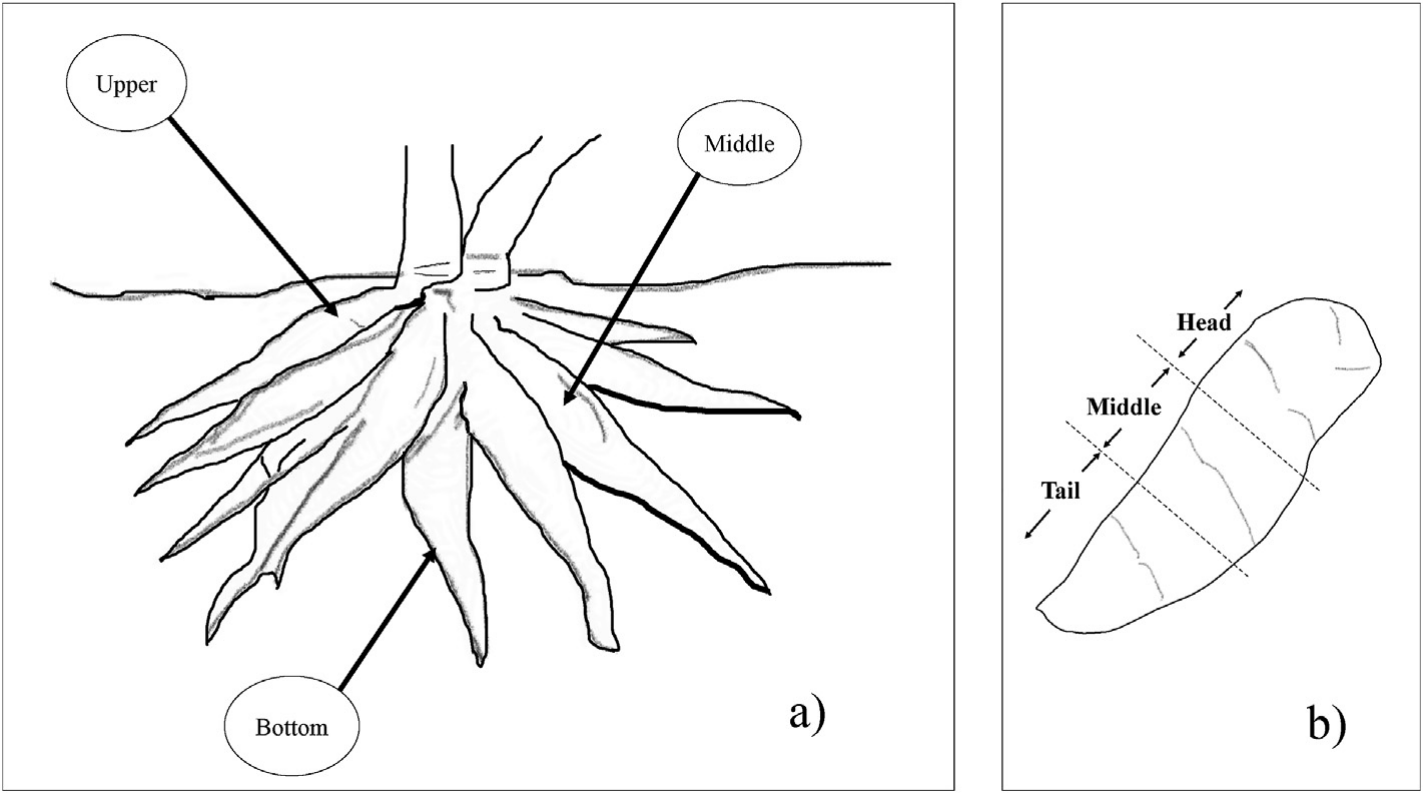
Sample collection used in this study. a) One tree collected three tubers. b) Each tuber separated into three section.

A total tuber of 76 cassava plants aged range from 4 to 12 months after planting (MAP) were collected from a breeding field, including the following varieties: Kasetsart 50 (KU50) (26 tubers), Rayong 9 (RY9) (7 tubers), Rayong 11 (RY11) (15 tubers), and CMR38-125-77 (28 tubers). The sample was collected from the harvest season 2019/2020. To select the tubers for this experiment, the most top tuber under the ground was selected, following middle and the tuber at the deepest position, respectively as show in Figure 1a). Each tuber was divided into three pieces and each piece was labelled as an individual sample as show in Figure 1b).

The samples were collected manually each week by human labourers. The four varieties of cassava were pulled up from underground at the same time (day by day). The numbers of cassava tubers for each variety were not the same. Samples were immediately sent to the laboratory at the Department of Agricultural Engineering, Khon Kaen University and were held at room temperature for one hour before any experiments were undertaken.

### 2.2 Measurement of specific gravity

**Figure 2:**
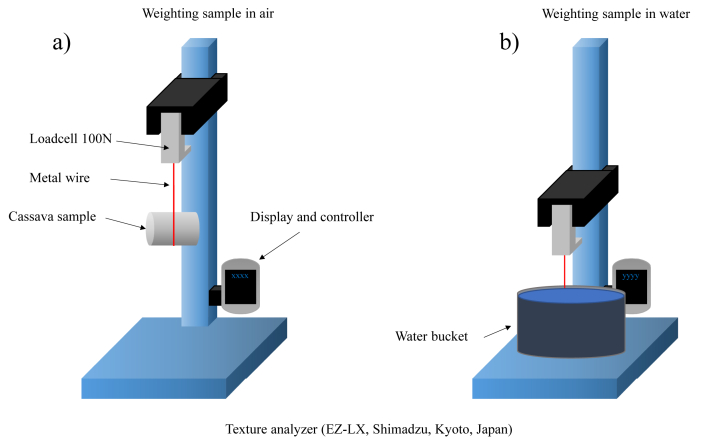
The schematic diagram of the cassava measurement system. a) Weighting sample in air b) Weighting sample in water.

The peel of sample was removed and the sample was weighed using digital scales with the resolution of 0.001g (AE-ADAM digital balance, Adam Equipment Inc, New York, USA). In the case of cassava, the specific gravity was more than that of water, therefore, the rope could be used. If the specific gravity of the sample was less than that of water, the hard stick was employed. To determine the SG, the sample was weighed in air and water using a texture analyser (EZ-LX, Shimadzu, Kyoto, Japan). Figure 2 shows a schematic diagram of the measurement of SC by using less sample. The SG can be calculated using Eq. (1):

(1) $$SG=\;\frac{W_{a}}{W_{a}-W_{w}}$$

where W_a_ and W_w_ are the weights of the sample in air and water, respectively (in mg).

### 2.3 Measurement of DM and SC

After measuring the weights in air and water, each sample was divided into two parts equally aimed to determine DM with duplicate. The value of DM for each part was determined using a hot air oven at 60 °C until the weight became constant. The dried sample was then placed in a desiccator for 30 min to cool down, and samples were weighed using a digital balance (AE-ADAM digital balance, Adam Equipment Inc, New York, USA, resolution of 0.001 g). The DM can be calculated using Eq. (2):

(2) $$DM\left(\%\right)=\frac{W_{f}}{W_{i}}\times 100$$

where W_i_ is the weight of the sample at the initial stage (%), W_f_ is the weight of the sample at the final stage (after drying) (g) and DM is the dry matter (%). After calculation, DM of two parts was averaged to one.

For determination of SC, two dried parts was then ground using an electronic blender (HR2115, Philips Pelumat, Dachten, Netherlands), mixed and sieved through a 180 mesh screen. After sieving, the ground samples were vacuum-packed into plastic bags and sent to the Cassava and Starch Technology Research Unit, National Center for Genetic Engineering and Biotechnology, National Science and Technology Development Agency, Thailand, for an analysis of the SC using the polarimetric method (ISO, 1997) of the European Economic Community. Each sample was done in duplicate. The SC was calculated using Eq. (3):

(3) $$SC\;\left(\%db\right)=\frac{\left[2000\times \left(P-P^{\prime }\right)\times 100\times L\right]}{\left[{\left(\alpha \right)}^{D}20\times 100\times M\right]}$$

where P is the total rotator power in degrees, P′ is the rotator power in degrees given by substances soluble in water, L is the standard tube length (200 mm), $\alpha^{D}$ is a specifies of the optical rotation of pure starch (where the value for cassava starch is 184^°^), and M is the moisture of the starch (%). Conversion of the SC from a dry basis to a fresh weight (%FW) can be done by multiplying the SC (%db) with the DM.

The standard error of laboratory (SEL) or repeatability of DM and SC were calculated. SEL was calculated as the standard deviation of differences between duplicate. SC and DM of each sample was done duplicate, then SEL was calculated as (Nie et al., 2009)

(4) $$SEL=\sqrt{\frac{\sum_{i}{\left(y_{1}-y_{2}\right)}^{2}}{N}}$$

where y_1_ − y_2_ is the difference between duplicate measurements by the reference method on sample i. N is the number of sample.

### 2.4 Empirical equation

After the values for the analyses were obtained, the relationships between SG and SC and between SG and DM were determined. The effects of the different sections of the sample (i.e. head, middle, and tail) and the level of the tuber in the soil (i.e. upper, middle and bottom) were studied. A multivariate analysis was carried out to find determined the empirical models.

The differences of the mean values for the SC and DM for four varieties, three different sections of the tuber, and three different levels of the tuber in the soil were analysed, using a one-way ANOVA, and compared using Duncan's multiple test with a statistical significance level of α = 0.05 using IBM SPSS Statistics v. 26.

### 2.5 Estimation of DM and SC for unknown samples

Unknown samples (23 samples from 14 tubers) were obtained from a different area and were used to test the performance of the empirical model. The samples were dug up from the ground and immediately sent to the laboratory to determine the values of SG, SC and DM in order to determine whether the developed empirical equation could be used to evaluate future samples (Nakawajana et al., 2018). The predicted values for the DM and SC were calculated as follows:

(5) $$Y_{pred}=a\times SG_{un}+b$$

where Y_pred_ denotes the predicted value for the DM or SC, and a and b denote the regression coefficient and the intercept of the empirical model, respectively. SG_un_ denotes the specific gravity of the unknown samples. The prediction performance for these unknown samples was measured using the standard error of prediction (SEP), and mean different (MD) which was calculated as follows:

(6) $$SEP=\sqrt{\frac{\sum {\left(Y-Y_{pred}\right)}^{2}}{n-1}}$$

(7) $$Mean\;different\;\left(MD\right)=\;\sum \frac{\left(Y-Y_{pred}\right)}{n}$$

where SEP, MD, Y, Y_pred_, and n denote the standard error of prediction, mean different, the measured value, the predicted value, and the number of samples, respectively.

## 3 Results and discussion

### 3.1 Statistical data on the SG, SC and DM of cassava tubers

Table 1 summarises the SG, SC and DM for individual cassava samples, including the maximum, minimum, average and standard deviation (SD) values. The values for the SG, SC calculated using the polarimetric method ranged between 26.68 and 44.99%FW, and therefore displayed a wide variability. A report from the Thai Tropical Development Institute (TTDI, 2000) states that the SC of cassava tubers can range from 25.87 to 41.88%FW, depending on the variety, age, surrounding environment and other factors (TTDI, 2000). These Figs were supported by the results reported by Montagnac et al. (2009), who found that the SC for fresh tubers was between about 32 and 35.00%FW at the mature stage. These results therefore cover the values of SC from the immaturity stage (4 MAP) to the harvesting maturity stage (between 8 and 12 MAP). The DM for individual sample ranged from 31.49 to 51.62%, and the values for SG were between 1.11 and 1.19. SEL denote precision of reference method show in Table 2.

**Table 1:** Statistical data on the SC and DM of cassava tubers.

Parameters	Sample number	Max	Min	Mean	SD
SG	174	1.19	1.11	1.16	0.012
SC by Polarimetric (%FW)	174	44.99	26.68	36.76	3.81
DM (%)	174	51.62	31.49	42.72	4.05

Max: Maximum value.

Min: Minimum value.

Mean: Averaging value.

SD: Standard deviation.

DM: Dry matter content.

SG: Specific gravity obtained from small sample size (1/3 of tuber length).

**Table 2:** Standard error of laboratory (SEL) of SC and DM in cassava samples.

Parameter	Mean of the different of duplicate	Standard error of laboratory (SEL)
SC (%FW)	0.11	0.34
DM (%)	0.85	1.70

SC: Starch content. DM: Dry matter content.

### 3.2 Relationships between SG, SC and DM content

Table 3 displays the statistical values for SC and DM for different varieties. The number of samples for each variety are not equal, since the weights of some samples were less than 100 g, for which the SC could not be measured using the polarimetric method. The results show that the mean values for the SC and DM of the RY9 and CMR38-125-77 varieties were significantly different, whereas those of KU50 and RY11 were similar. The values for the SC and DM for RY11 and KU50 were the highest, followed by CMR38-125-77 and RY9. However, the standard deviation for SC and DM was around 2–4%, depending on the variety. The results therefore show that RY11 gave the highest values for SC and DM from the group.

**Table 3:** The characteristics of SC and DM of the cassava tubers.

Varieties	Sample number	SC (%FW)				DM (%)			
		Max	Min	Mean	SD	Max	Min	Mean	SD
CMR38-125-77	64	40.25	28.95	34.08b	2.86	45.89	34.41	39.80b	3.02
KU50	62	44.99	33.30	39.12a	2.50	51.62	40.42	45.19a	2.62
RY11	33	43.40	34.29	39.66a	1.89	49.17	41.21	45.94a	1.72
RY9	15	36.14	26.68	32.11c	2.62	42.56	31.49	37.95c	3.11

Different letters in the same column within a variety indicates the different means that are significant at p > 0.05 by the Duncan's multiple range test. SC is starch content. DM is dry matter content.

A report from Reinhardt Howeler (2014) described the RY11 variety of cassava as being characterised by high SC and high yield production. RY9 has the characteristic properties of high yield, high SC and high ethanol yield; this variety was specifically developed for the production of ethanol, as the starch has an unusually high conversion rate to ethanol Reinhardt Howeler (2014). Meanwhile, the KU50 variety was characterised by high yield and high DM.

Table 4 show more details of the SC and DM for each variety, for different sections of the tuber (i.e. head, middle and tail) and different levels of the tuber in the soil (i.e. upper, middle and bottom). The results show that the mean values of different tuber section for the SC and DM of the RY9 and RY11 varieties were not significantly different at 95% confidence level, meaned that the different sections of the tuber had no influence on SC and DM for both varieties. Meanwhile, the results for KU50 and CMR38-125-77 were not different, meaned that the sections of the tuber influenced the SC and DM value. The most of SC and DM of head and middle section of every variety was not different, and tail section was not different. Therefore, the tuber sections at either head or middle could be used as representative tuber (whole tuber).

**Table 4:** Statistical data of SC and DM for different varieties.

Varieties	Section	SC (%FW)		DM (%)		Position	SC (%FW)		DM (%)	
		Mean	SD	Mean	SD		Mean	SD	Mean	SD
KU50	Head	39.67a	2.72	46.08a	2.75	Upper	39.97a	2.28	46.16a	2.77
	Middle	39.14a	2.57	45.02ab	2.52	Middle	39.49a	2.26	45.34ab	2.78
	Tail	38.28a	1.94	44.07c	2.18	Bottom	37.22b	3.24	43.3b	3.59
CMR38-125-77	Head	35.60a	2.29	41.74a	2.23	Upper	35.68a	2.77	41.52a	3.25
	Middle	33.91ab	2.8	39.59b	2.79	Middle	34.1ab	3.62	40.14ab	3.66
	Tail	32.45b	3.06	37.81c	3.15	Bottom	32.76b	2.43	38.45b	2.58
RY11	Head	39.94a	1.74	46.38a	1.16	Upper	39.56a	0.79	45.66a	0.57
	Middle	39.72a	2.2	45.81a	1.79	Middle	38.35a	3.15	44.88a	3.31
	Tail	38.66a	1.36	44.96a	1.7	Bottom	38.63a	1.68	44.76a	2.04
RY9	Head	32.95a	1.39	38.99a	1.85	Upper	33.29a	2.4	39.99a	2.22
	Middle	31.74a	2.9	37.40a	3.42	Middle	33.51a	0.88	39.05b	1.37
	Tail	31.63a	3.5	37.45a	4.08	Bottom	35.23b	3.51	39.12b	4.42

Different letters in the same column within a variety indicates the different means that are significant at p > 0.05 by the Duncan's multiplerage test. SC is starch content. DM is dry matter content.

In term of the levels of the tuber in the soil (upper, middle and bottom), it had a slightly influence on the SC and DM value for KU50, CMR38-125-77 and RY9 varieties and there had no affect to the SC and DM for the RY11 variety (SC and DM value of the tubers in the different soil levels were no significantly different at 95% confidence level). The most of SC and DM for upper and middle level tuber in the soil was not different.

In the opinion of the authors, the tuber at the either upper or middle levels in the soil can be used as representative cassava trunk (whole trunk). It is beneficial to make the process easier to collect samples for evaluation due to the upper level is easy for sampling.

**Figure 3:**
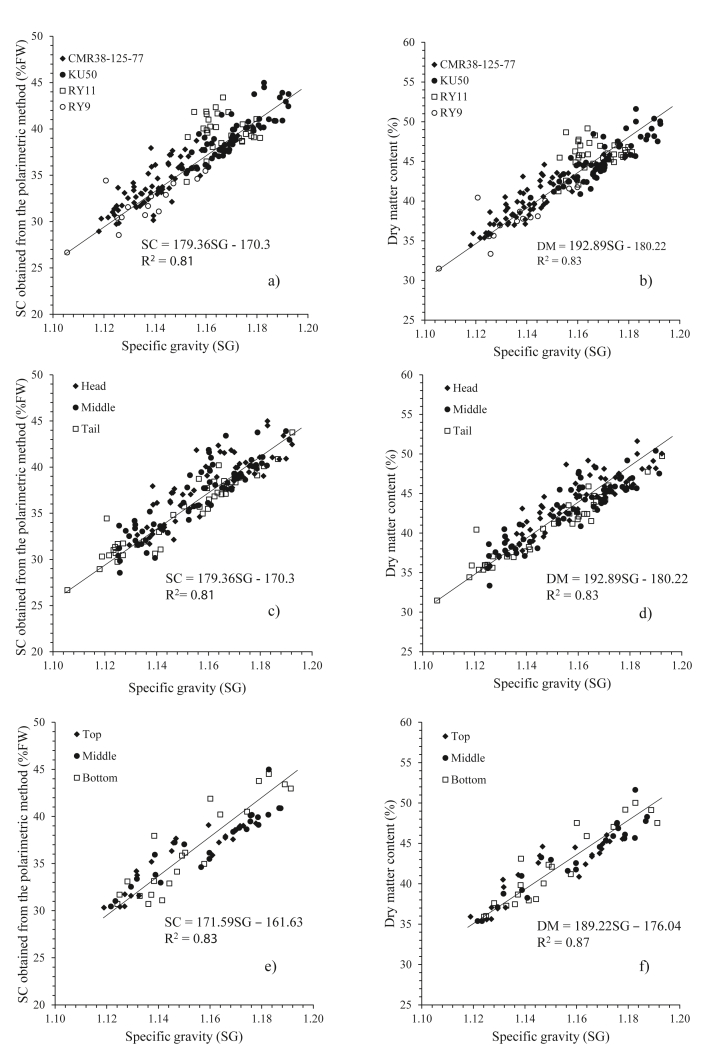
Relationship between SG and SC and DM of cassava roots. a) SG and SC from different four varieties. b) SG and DM from different four varieties. c) SG and SC from different sections of tuber. d) SG and DM from different sections of tuber. e) SG and SC from different tuber level f) the relationship between SG and DM from different tuber level.

Figure 3 shows the relationships for all samples, between the SG and SC, and between the SG and DM, based on the different varieties, the section of the tuber (head, middle and tail) and the level of the tuber in the soil (upper, middle and bottom). These Figs show the positive correlation between SC and DM with the SG. Similarly to the results reported by Vanasse et al. (1951), they found that the SG correlated to the eating quality of the potatoes in terms of their edibility. This results therefore was one example to demonstrate that the SG could be used to measure the internal quality of the cassava tubers.

Table 5 shows the results of full factorial experiment of SC from individual variety to expect the main and interaction effect between tuber position and section. The results show that section, position and section∗position did not have a significant impact on SC for the RY11 and RY9 varieties. This means that the samples obtained from RY11 and RY9 could be used for building the empirical equations due to these have not significantly difference. For CMR38-125-77 variety, the position and section∗position did not have a significant impact, while section have a significant impact on SC. Although the KU50 was completely significanted, mean that the position and section had an interaction, moreover main effect of position and section was significance. Table 6 exhibited results of DM from full factorial ANOVA. The result show position, section and position∗section have a significant effect on DM for KU50. Meanwhile, position and position∗section have a significant effect on DM for RY11. For CMR38-125-77, there have no interaction between position and section, while main effect was sinificane. On the contrary, RY9 were not significant in position, section and position∗section, respectively.

**Table 5:** Results of SC from full factorial ANOVA.

Variety	Source	Type III Sum of Squares	df	Mean Square	F	Sig.
CMR38-125-77	Position	0.555	2	0.278	0.129	0.880
	Section	112.574	2	56.287	26.084	0.000
	Position ∗ Section	10.255	4	2.564	1.188	0.329
	Error	97.107	45	2.158		
	Total	62226.992	54			
KU50	Position	71.887	2	35.943	27.719	0.000
	Section	44.753	2	22.376	17.256	0.000
	Position ∗ Section	56.275	4	14.069	10.850	0.000
	Error	53.165	41	1.297		
	Total	79000.896	50			
RY11	Position	0.192	2	0.096	0.060	0.942
	Section	1.014	2	0.507	0.319	0.735
	Position ∗ Section	0.820	4	0.205	0.129	0.968
	Error	14.317	9	1.591		
	Total	28218.676	18			
RY9	Position	26.170	2	13.085	1.437	0.265
	Section	26.636	2	13.318	1.463	0.259
	Position ∗ Section	30.310	4	7.578	0.832	0.523
	Error	154.762	17	9.104		
	Total	32232.982	26			

df: Degree of freedom.

Sig.: Significant.

**Table 6:** Results of DM from full factorial ANOVA.

Variety	Source	Type III Sum of Squares	df	Mean Square	F	Sig.
CMR38-125-77	Position	17.329	2	8.664	4.916	0.012
	Section	283.607	2	141.803	80.455	0.000
	Position∗ Section	16.019	4	4.005	2.272	0.076
	Error	79.313	45	1.763		
	Total	84701.126	54			
KU50	Position	127.023	2	63.511	52.426	0.000
	Section	89.985	2	44.993	37.139	0.000
	Position ∗ Section	70.113	4	17.528	14.469	0.000
	Error	49.670	41	1.211		
	Total	104032.722	50			
RY11	Position	5.052	2	2.526	18.316	0.001
	Section	0.224	2	0.112	0.811	0.474
	Position ∗ Section	6.467	4	1.617	11.723	0.001
	Error	1.241	9	0.138		
	Total	38667.959	18			
RY9	Position	22.036	2	11.018	0.837	0.450
	Section	48.398	2	24.199	1.839	0.189
	Position ∗ Section	39.250	4	9.812	0.746	0.574
	Error	223.740	17	13.161		
	Total	42820.133	26			

df: Degree of freedom.

Sig.: Significant.

Table 7 shows the overall coefficient of determination (R^2^) and regression equations for the SG, SC and DM for an individual part of a cassava sample. The table shows that a simple linear regression equation can be used to estimate the SC and DM if the SG for a cassava tuber is known. The range of R^2^ was between 0.03 and 0.95. The empirical equations which were developed from each variety, it provided R^2^ around 0.70–0.80. The RY11 variety showed a poor R^2^, with values of 0.03 for the SC and 0.06 for the DM, and this might indicate that it was not suitable for that particular area. This variety also required a great amount of water (DOA, 2020), but this experiment was measured during the dry season. This reason might impact to the cassava tubers, it was tiny and blighted. The SG for all samples was highly related to the SC and DM, with values of R^2^ of 0.81 and 0.83, except the vareity of RY11, respectively.

**Table 7:** Overall coefficient of determination (R^2^) and empirical equations rerated SG to SC, and DM of cassava tuber.

	Sample	Parameters	R^2^	Regression equations
Total	Combined sample	SG vs SC	0.81	SC = 179.36SG - 170.3
	Combined sample	SG vs DM	0.83	DM = 192.89SG - 180.22
Variety	CMR38-125-77	SG vs SC	0.74	SC = 173.39SG -163.69
	CMR38-125-77	SG vs DM	0.79	DM = 188.66SG-175.40
	KU50	SG vs SC	0.74	SC = 187.75SG - 180.58
	KU50	SG vs DM	0.71	DM = 193SG - 180.64
	RY11	SG vs SC	0.03	SC = 35.33SG - 1.56
	RY11	SG vs DM	0.06	DM = 48.43SG - 10.56
	RY9	SG vs SC	0.66	SC = 138.80SG - 125.71
	RY9	SG vs DM	0.70	DM = 170.42SG-155.82
Section	Head	SG vs SC	0.80	SC = 175.76SG - 166.27
	Head	SG vs DM	0.82	DM = 188.21SG-174.70
	Middle	SG vs SC	0.82	SC = 183.51SG - 175.39
	Middle	SG vs DM	0.84	DM = 198.02SG - 186.24
	Tail	SG vs SC	0.80	SC = 186.50SG - 178.91
	Tail	SG vs DM	0.83	DM = 201.55SG - 190.39
Position	Upper	SG vs SC	0.89	SC = 168.35SG - 158.16
	Upper	SG vs DM	0.93	DM = 189.11SG - 176.25
	Middle	SG vs SC	0.90	SC = 175.70SG - 166.80
	Middle	SG vs DM	0.94	DM = 197.03SG - 185.56
	Lower	SG vs SC	0.92	SC = 182.08SG - 174.27
	Lower	SG vs DM	0.95	DM = 202.94SG - 192.47

SG: specific gravity.

SC: starch content obtained from the polarimetric method.

DM: dry matter content obtained from dry oven method.

**Figure 4:**
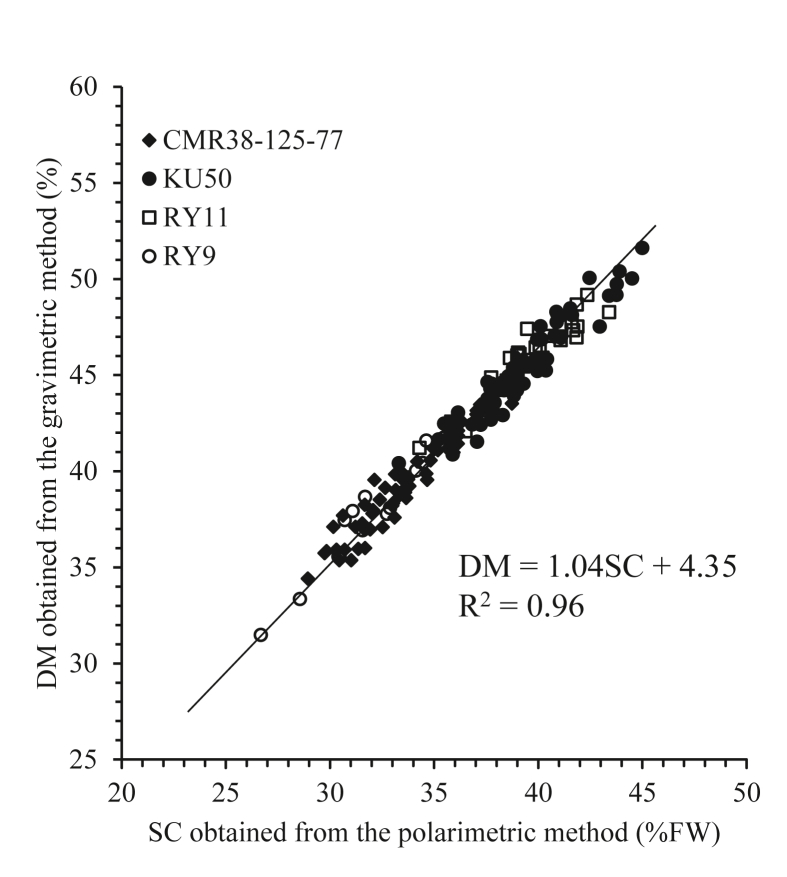
The relationship between cassava SC and DM of cassava.

Figure 4 shows a scatter plot of SC versus DM. The SC was also strongly correlated with the DM, with a value for R^2^ of 0.96. These results were similar to those reported by Vanasse et al. (1951), who studied the factors affecting the internal quality of potatoes. Their SG showed a very strong correlation with the DM, and the size of the potato tubers did not affect the SG.

### 3.3 Estimation of SC and DM for unknown samples

When the empirical equations had been developed, they were validated using unknown samples. The unknown samples were used to determine the SG, which was assigned as the independent variable to estimate the SC and DM using the empirical equations in Table 7.

The measured and predicted values of SC and DM for the unknown samples, predicted using individual and combined models, are shown in Table 8. The individual models were taken from Table 7, and the equations were chosen from the samples which developed from tuber position due to their provided higher R^2^ when compared to another equation among the table. The results of these individual equations provided the MD and SEP for the SC and DM, with values of -0.23%FW, 1.05%FW and for DM were -0.36%FW and 3.51%, respectively. The results of the combined equation gave better results than those of the individual equations, with values for MD and SEP for the SC were -0.66%FW, 1.03%FW and for DM were -0.89%FW, 3.49%, respectively. These results indicated that the values of the SEP for the SC and DM of the unknown samples, which were representative samples for the future planting, were no greater than 1.3%FW and 3.49%. Even though the individual equation provided higher R^2^ than the combined model, but combined model also gave higher accuracy (lowest SEP when validated by unknown sample) than that of individual equation. The authors suggested that the models applicators for measuring the SC and DM of fresh cassava could apply these equations (combine models), which were built on the polarimetric method and dry oven method. For practical purposes, the authors also suggested that the combined equations could be applied by such as cassava breeders and farmers, due to its easy application and relatively high accuracy.

**Table 8:** Measured versus predicted value of unknown samples in prediction of SC and DM of cassava tubers.

Sample			SC (%FW)					DM (%)				
Varieties	Section	Position	Measured value	Individual model		Combined model		Measured value	Individual model		Combined model	
				Predicted value	Different	Predicted value	Different		Predicted value	Different	Predicted value	Different
CMR38-125-77	Head	Upper	31.14	30.83	0.30	31.05	0.08	35.70	36.05	-0.34	36.32	-0.62
CMR38-125-77	Head	Upper	29.74	29.11	0.64	29.21	0.53	35.05	34.11	0.94	34.34	0.71
CMR38-125-77	Head	Upper	29.24	30.34	-1.10	30.53	-1.29	34.30	35.49	-1.20	35.76	-1.46
CMR38-125-77	Head	Middle	29.10	30.01	-0.91	29.66	-0.55	34.92	34.10	0.82	34.82	0.10
CMR38-125-77	Middle	Lower	28.70	29.69	-0.99	30.61	-1.91	34.61	34.86	-0.24	35.85	-1.23
CMR38-125-77	Middle	Upper	29.25	30.03	-0.78	30.20	-0.94	34.06	35.15	-1.08	35.40	-1.34
CMR38-125-77	Middle	Upper	29.81	31.66	-1.85	31.94	-2.12	34.00	36.98	-2.98	37.27	-3.27
CMR38-125-77	Middle	Upper	28.93	32.07	-3.14	32.37	-3.44	34.29	37.44	-3.15	37.74	-3.45
CMR38-125-77	Tail	Middle	30.19	29.84	0.36	30.43	-0.24	34.20	34.95	-0.75	35.65	-1.45
CMR38-125-77	Tail	Middle	23.86	24.12	-0.26	24.59	-0.73	35.15	28.53	6.62	29.38	5.77
CMR38-125-77	Tail	Lower	29.86	29.74	0.12	30.66	-0.80	37.01	34.91	2.10	35.90	1.11
KU50	Head	Middle	28.33	29.19	-0.85	29.77	-1.44	33.57	34.22	-0.65	34.94	-1.37
KU50	Head	Lower	23.10	23.20	-0.10	24.22	-1.12	32.97	28.12	4.85	28.97	4.00
KU50	Head	Lower	28.88	28.04	0.84	28.99	-0.11	34.03	33.36	0.67	34.10	-0.07
KU50	Middle	Upper	28.58	29.88	-1.31	30.04	-1.46	36.11	34.98	1.13	35.24	0.87
KU50	Middle	Upper	29.24	29.68	-0.45	29.83	-0.59	37.01	34.76	2.25	35.01	2.01
KU50	Middle	Middle	30.57	30.55	0.01	31.17	-0.60	26.79	35.75	-8.96	36.44	-9.65
KU50	Tail	Lower	31.81	31.67	0.14	32.56	-0.75	33.74	37.06	-3.32	37.95	-4.20
RY11	Head	Middle	30.04	28.43	1.61	29.00	1.04	35.34	33.37	1.96	34.11	1.22
RY11	Head	Middle	35.38	35.09	0.29	35.79	-0.41	32.47	40.84	-8.36	41.42	-8.95
RY9	Head	Upper	31.00	30.08	0.92	30.26	0.75	35.98	35.21	0.77	35.46	0.52
RY9	Head	Upper	31.55	30.83	0.71	31.05	0.49	34.88	36.05	-1.17	36.32	-1.45
RY9	Middle	Upper	30.29	29.76	0.53	29.90	0.38	36.74	34.84	1.90	35.09	1.65
				**MD**	-0.23		-0.66		**MD**	-0.36		-0.89
				**SEP**	1.05		1.03		**SEP**	3.51		3.49

MD: mean different.

SEP: standard error of prediction.

### 3.4 Comparison with existing methods

Wholey and Booth (1979) studied the relationship between the SG and the conventional method with a Reimann balance, which was used as an industrial standard determination of SC, and found that the SG was strongly related to both the SC and DM. They then created an empirical equation based on the relation between the SG and SC, which was obtained using the Krochmal and Kilbride (1996) method. Several breeders have used their equation to estimate the SC for cassava tubers, although this equation requires a sample of at least 5 kg.

For this reason, cassava at the end of harvesting stage (12 months after planting, 12 MAP) was gathered manually, due to the need to obtain a sample that was large enough to measure the SC (Santisopasri, 1998). The SG method developed by Wholey and Booth (1979) was then applied in various industries, and the calculated results were SC = 159.1SG−147.0 and DM = 142.3SG−124.9, respectively.

**Figure 5:**
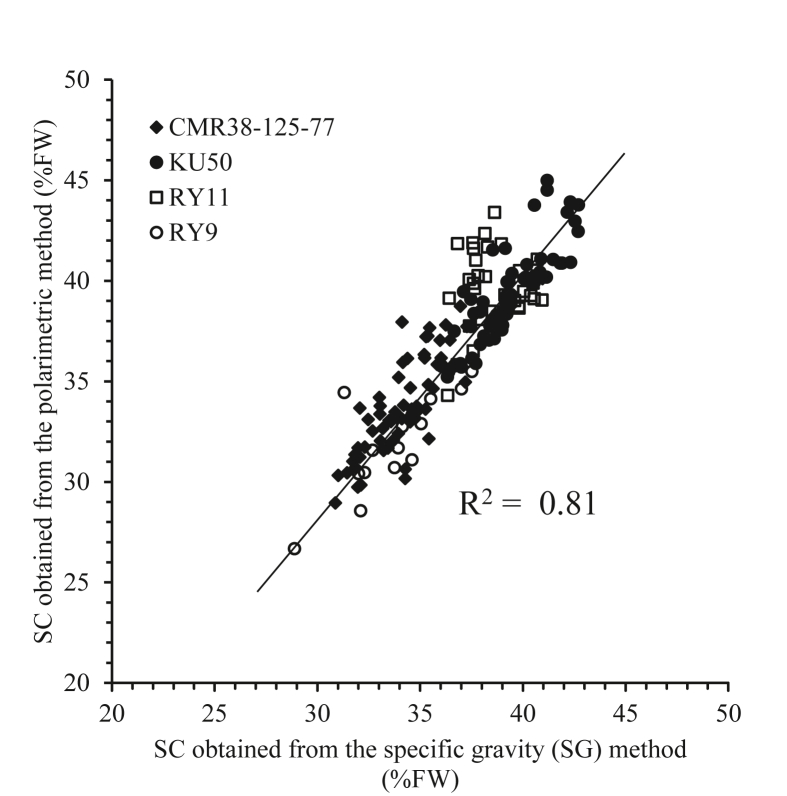
The scatter plot of cassava SC between polarimetric method and SG method.

Figure 5 shows a scatter plot of the values of SC obtained by the polarimetric method and the values for SC obtained from the SG method (i.e. Wholey and Booth's equation). Although the values from both methods displayed a good relationship, those obtained from the SG method were slightly higher than for the polarimetric method. Bantajan and Rittiron (2016) succeeded in using NIR spectroscopy to predict the SC for cassava tubers, and showed that the differences in the results of SC measurements using the SG and polarimetric methods were not greater than 6.2%. Furthermore, this study proved that the SG method could provide a value of SC that was higher than that from the polarimetric method.

These results were supported by Santisopasri (1998), who found that the highest SC was obtained for the root when harvested at 8 MAP. In contrast, Sriroth et al. (1999) found that the SC reached a maximum at 14 MAP. However, in general, there is an increase in SC in the dry season and a decrease in the wet season, since starch is used to create new leaves and branches. Hence, the highest SC obtainable depends on the harvesting period (Buddhakulsomsiri et al., 2015, 2018).

Breeders consider key information on the highest SC, DM and HI for harvesting, and can use these indexes to evaluate productivity. However, although breeders can obtain this information, they cannot identify changes in it until the harvesting process (4–12 MAP) is complete, since some varieties cannot provide sufficient weights for monthly measurements (Maraphum et al., 2020).

Ikeogu et al. (2017) reported that the values of the DM for cassava obtained by the oven-drying and SG methods showed a moderate correlation (r = 0.49). The DM could be estimated using the equation DM = 158.3SG-142.0, which provided a coefficient of determination (R^2^) of 0.84. This gave values for R and R^2^ of 0.91 and 0.83, indicating that the relationships between SG and DM in the present work and previous studies by Fukuda et al. (2010) and Kawano et al. (1987) are similar.

Our results were also compared with another dataset, provided by the National root crops research institute (NRCRI), which used an equation of DM = 67.33SG−37.03 and gave a value of R^2^ = 0.23. Our results gave a higher value for R^2^ than the NRCRI data. The SG method can therefore be said to be strongly related to the percentages of SC and DM in cassava samples.

Moreover, this method was developed on a small sample (pieces of cassava) from the main variety in a breeding program. Besides, this method also used from young sample to old sample (harvesting stage), which certain that the method and results of this study could be used to investigate the internal quality of future samples during breeding.

When used in a breeding programme, authors suggested that when measuring the SC and DM of cassava, the samples should be fresh, since otherwise they may float in water, meaning that the SC cannot be measured. Our equations for evaluating the SC and DM show that the SG can be estimated based on the SC and DM. However, this method involves measuring the SC on a wet basis; if breeders need to know the SC for cassava on a dry basis, the polarimetric method must still be used.

## 4 Conclusion

This study presents an approach that uses a smaller sample of cassava for the measurement of the SC and DM with the determination of SG, where the SG obtained from small sample size (~100 g). The SG was strongly correlated with the SC and DM, with values for the coefficient of determination (R2) of 0.81 and 0.83, respectively. The most effective empirical equation was tested using unknown samples, provided the mean different of -0.66%FW, -0.89% and standard error of 1.02%FW and 3.49% for the SC and DM, respectively. The accuracy was high, which was acceptable with useable in most application. Therefore, the empirical equation could be used as an alternative method to the polarimetry and dry oven.

In terms of the section tuber and level of the tuber in the soil, the SC and DM were highest in the head section and the upper level. Author suggested that measurement of the SC and DM at the middle section of tuber which collected from the either or middle level in the soil can be used as representative SC and DM value of whole trunk. The combined empirical equation is the best choice for measuring the SC and DM. In a real-world situation, the upper levels of the tuber are more suitable for measuring the quality of the cassava, since it is easy to obtain the samples. In some cases, it is difficult to obtain lower tubers due to their very deep position.

This method could be offered an alternative for breeders, as it is rapidly increasing the possibility of discovering a valuable new variety with lower costs and increase the time in the operation process. Thus, the SC can be estimated by SG for freshly harvested samples, thus eliminating the need to prepare samples and send them to a laboratory for measuring by the polarimetric method. By the SG method and reducing errors due to loss of SC in the sample arising from the delay between harvesting and SC determination. In addition, this approach could help to address the challenges of new methods which improving the overall quality of phenotyping for cassava.

## Declarations

### Author contribution statement

Kanvisit Maraphum: Conceived and designed the experiments; Performed the experiments; Analyzed and interpreted the data; Wrote the paper.

Khwantri Saengprachatanarugt: Analyzed and interpreted the data; Contributed reagents, materials, analysis tools or data.

Seree Wongpichet; Panmanas Sirisomboon: Analyzed and interpreted the data.

Arthit Phuphuphud: Performed the experiments.

Jetsada Posom: Conceived and designed the experiments; Contributed reagents, materials, analysis tools or data; Wrote the paper.

### Funding statement

This work was supported by the 10.13039/501100004192National Science and Technology Development Agency(NSTDA), RD&E Funding Contract (FDA-CO-2562-10130-TH), Thailand; Faculty of Engineering, Khon Kaen University, Thailand; the Research EN KKU; Research and Graduate Studies Khon Kaen University and Applied Engineering for Important Crops of the North East research group, Khon Kaen University.

### Data availability statement

Data included in article/supplementary material/referenced in article.

### Declaration of interests statement

The authors declare no conflict of interest.

### Additional information

No additional information is available for this paper.
